# Green Fabrication of Zinc-Based Metal–Organic Frameworks@Bacterial Cellulose Aerogels via In Situ Mineralization for Wastewater Treatment

**DOI:** 10.3390/molecules30050982

**Published:** 2025-02-20

**Authors:** Xinru Liu, Jie Gu, Yongqi Cao, Liping Tan, Tongjun Liu

**Affiliations:** 1 Shandong Provincial Key Laboratory of Microbial Engineering, Department of Bioengineering, Qilu University of Technology, Shandong Academy of Sciences, Jinan 250353, China; 10431221262@stu.qlu.edu.cn (X.L.); gujie0218@163.com (J.G.); 10431240818@stu.qlu.edu.cn (Y.C.); 2 Guangxi Key Laboratory of Clean Pulp and Papermaking and Pollution Control, College of Light Industry and Food Engineering, Guangxi University, Nanning 530004, China

**Keywords:** metal–organic framework, bacterial cellulose, adsorption, wastewater

## Abstract

Compared to conventional adsorbents, zinc-based metal–organic frameworks (MOFs) such as zeolite imidazolium skeleton-8 (ZIF-8) exhibit enhanced thermal, chemical, and structural stability. Nonetheless, their powdered form results in limited dispersibility in aqueous solutions and a tendency to aggregate, which significantly restricts their utility in adsorption applications. This study reports a green composite aerogel through the in situ mineralization of ZIF-8 onto bacterial cellulose (BC) for the effective removal of toxic metal ions (Cu^2+^) and Congo red (CR) from wastewater. The ZIF@BC composite aerogel was characterized using scanning electron microscopy, Fourier transform infrared spectroscopy, thermogravimetric analysis, X-ray diffraction, X-ray photoelectron spectroscopy, and specific surface area analysis. The findings indicated that the ZIF-8 produced were evenly distributed across the BC nanonetwork, facilitating effective adsorption of CR and Cu^2+^. The maximum adsorption capacities of the ZIF@BC aerogels were determined to be 397.55 mg/g for CR and 424.80 mg/g for Cu^2+^, as per the Langmuir isotherm. Furthermore, the ZIF-8@BC aerogels demonstrated excellent selectivity and reusability, particularly for CR adsorption. The proposed mechanism for the interaction between the composite aerogel and CR and Cu^2+^ involves electrostatic interactions, hydrogen bonding, π-π bonding, coordination bonding, ion exchange, microchemical precipitation, and pore diffusion. This research offers significant promise for the utilization of MOF powders and highlights substantial industrial potential.

## 1. Introduction

Water serves as the fundamental resource for human survival and is vital to both human beings and ecosystems. However, the advancement of industrialization has led to significant discharges of industrial wastewater containing pollutants into natural water bodies [1,2]. This not only leads to water pollution but also poses a substantial threat to human survival. Addressing the treatment of industrial wastewater has become an urgent issue that requires resolution. Among them, dyes and toxic metal ions have strong stability, high permeability, and high toxicity, which can cause irreversible harm to human and animal health [3,4]. Thus, the effective removal of dyes and toxic metal ion pollutants from wastewater is essential.

Various methods have been developed to address dye and toxic metal pollution, including membrane filtration [5], electrochemical methods [6], and biological treatment techniques [7]. Among the various methods, adsorption is regarded as a particularly effective decontamination technology because of its simplicity, cost-effectiveness, availability of adsorbents, and high removal efficiency [8,9,10]. Traditional adsorbents primarily consist of organic or inorganic porous materials [11], including zeolite [12], activated carbon [13], graphene [14], and carbon nanotubes [15]. However, these materials present certain disadvantages when it comes to pollutant adsorption. For instance, zeolites exhibit low efficiency in adsorbing anions and organic compounds [16]. While activated carbon offers a large surface area and favorable pore structure, it is associated with high costs, low reversible adsorption capacity, and challenges in regeneration [17,18]. Layered graphene has low porosity and a tendency to aggregate in aqueous environments [19]. Additionally, carbon nanotubes exhibit strong interactions and are prone to aggregation, which decreases their specific surface area [20].

Metal–organic frameworks (MOFs) are an emerging category of porous structures formed by the interaction and coordination of metal ions or clusters with organic molecules, thereby forming highly organized networks. MOFs possess remarkable characteristics, including high specific surface area, elevated porosity, flexible structures, and excellent catalytic activity [21,22], making them demonstrate outstanding capabilities in adsorbing dyes and toxic metal ions, such as ZIF-8, ZIF-67, ZIF-L, etc. [23]. Among numerous MOFs, Zn-based MOFs have attracted much attention due to their various advantages, such as adjustable physical and chemical properties, high thermal stability, high chemical stability, excellent water stability, and rich synthesis strategies. However, the predominant form of MOFs as microcrystals limits their recycling, processing, and application potential [24].

Bacterial cellulose (BC) is a three-dimensional network formed by hydrogen-bonded assemblies of hydroxyl groups (-OH), exhibiting a honeycomb-like layered structure perpendicular to the gas–liquid interface. As a renewable resource, BC has garnered considerable attention due to its high purity, crystallinity, elastic modulus, tensile strength, tear resistance, and biocompatibility. Its chemically modified surface and unique optical properties have also attracted significant interest [25,26]. BC serves as an ideal biological polymer, as its surface-rich hydroxyl groups can facilitate the mineralization of inorganic materials through physical adsorption, covalent bonding, and surface graft polymerization reactions, thereby enabling the successful preparation of various nanocellulose-based inorganic composite materials and significantly enhancing the functional characteristics of these composites. Currently, a series of inorganic materials with unique properties are mineralized onto the surface of BC, including metal nanoparticles [27], oxide nanoparticles [28], inorganic salts [29], and carbon materials [30]. BC offers high porosity and structural support to composite aerogels, reducing the aggregation of individual MOF nanoparticles and improving their recoverability.

In this work, BC was used to synthesize (as shown in Figure S1) a highly adsorptive, selective, and reusable zinc-based MOF (ZIF-8) multipore aerogel adsorbent in situ using its BC mineralization ability, resulting in the construction of a ZIF-8@BC aerogel. The synthesized ZIF-8@BC aerogel was prepared in situ using a simple green method without employing organic solvents, which can quickly and effectively remove CR and Cu^2+^ in wastewater.

## 2. Materials and Methods

### 2.1. Materials

BC was acquired from Guilin Qihong Technology Co., Ltd. (Guilin, China). NaHSO_3_ and 2-methylimidazole (2-MI) were sourced from Aladdin Biochemical Technology Co., Ltd. (Shanghai, China). NaCl and NaOH were obtained from Sangon Biotech Co., Ltd. (Shanghai, China). *tert*-Butanol, NaNO_3_, NaHCO_3_, Zn(NO_3_)_2_·6H_2_O, and CuSO_4_·5H_2_O were procured from Sinopharm Chemical Reagent Co., Ltd. (Shanghai, China). Congo red (CR, Mw = 696.68 g/mol) was obtained from Tianjin Kemiou Chemical Reagent Co., Ltd. (Tianjin, China).

### 2.2. Methods

#### 2.2.1. Fabrication of ZIF-8@BC-X

The ZIF-8@BC-X composite aerogel was synthesized through in situ methods, with the preparation process illustrated in Figure S1. Initially, purified BC was cut into small pieces (about 1 cm × 1 cm) then immersed in a *tert*-butanol solution while being magnetically stirred for 5 h to replace the water. The resulting BC was then freeze-dried. After that, BC (0.1 g) was added to deionized water (40 mL), along with the addition of Zn(NO_3_)_2_·6H_2_O based on the amounts specified in Table S1 to create solution A. An equivalent amount of 2-MI was dispersed separately in deionized water (40 mL) to form uniform solution B. Finally, both solutions were thoroughly mixed, magnetically stirred for 2 h, and allowed to stand for 22 h. After washing with deionized water and replacing the water with *tert*-butanol, the resulting sample was freeze-dried and designated as ZIF-8@BC-X (as shown in Table S1).

#### 2.2.2. Adsorption Experiment

First, 0.01 g of ZIF-8@BC-X was added to CR solution (20 mL) at a concentration of 500 mg/L (and 200 mg/L copper sulfate). Subsequently, the prepared mixture was allowed to react at 25 °C for 24 h and 180 r/min in a thermostatic oscillator (MQD-B3R, Minquan, Shanghai, China). The CR solution concentration was determined with a UV spectrophotometer (UV-6100, Metash, Shanghai, China) at 500 nm, while the Cu^2+^ concentration was obtained using an inductively coupled plasma emission spectrometer (ICP-OES 5110, Agilent, Santa Clara, CA, USA). The adsorption capacities of CR and Cu^2+^ were calculated according to our previous work [31]. To evaluate the reusability and selective adsorption of ZIF-8@BC-X, the following protocol was used: the reacted ZIF-8@BC-X was placed in a 1 M NaOH solution to facilitate desorption and regeneration, followed by washing with deionized water until neutral, preparing it for further reuse cycles. To evaluate the impact of coexisting ions on the removal potential of contaminants, a CR solution (200 mg/L) of mixed ions (NO_3_^−^, HSO_3_^−^, HCO_3_^−^, Cl^−^) was prepared and used for adsorption experiments. Each adsorption test was conducted with three replicates to ensure reliability.

The impact of pH was determined on the adsorption behavior of CR and Cu^2+^ within the pH ranges of 3.0–10.0 and 2.0–6.0, respectively. The isothermal adsorption curve was obtained under optimal adsorption pH conditions, employing CR and Cu^2+^ concentrations of 50–1000 mg/L and 50–1500 mg/L, respectively. The isothermal adsorption curve was analyzed via two isothermal models, the Langmuir and Freundlich models [32].

To investigate adsorption kinetics, experiments were performed at optimal pH levels, using a concentration of 1000 mg/L for either CR or Cu^2+^ solution. The adsorption mechanism of the ZIF-8@BC-X aerogel was analyzed using both the pseudo-first-order (PFO) as well as pseudo-second-order (PSO) kinetic models for fitting the obtained data and evaluating the associated kinetic parameters [32].

### 2.3. Characterization

The morphology along with the structure of BC and ZIF-8@BC-X were examined using scanning electron microscopy (SEM, Regulus8220, Hitachi, Tokyo, Japan). Specific surface areas of BC and ZIF-8@BC-X were assessed with a surface analyzer (BET, Micromeritics ASAP 2460, Micromeritics, Norcross, GA, USA). The crystal states of BC and ZIF-8@BC-X were evaluated through X-ray diffraction (XRD, Smartlab SE, Rigaku, Tokyo, Japan) at a speed of 20°/min, an angle range of 5–80°, along with 0.02° step width. The functional groups present in BC and ZIF-8@BC-X were recognized using Fourier transform infrared spectroscopy (FTIR, Spectrum Two, PerkinElmer, Waltham, MA, USA) over a range of 400–4000 cm^−1^. Assessment for the thermal stability was carried out with the help of thermogravimetric analysis (TGA, TGA 4000, PerkinElmer, MA, USA) across a temperature range of 30–800 °C. X-ray photoelectron spectroscopy (XPS) was carried out using an ESCALAB Xi+ photoelectron spectrometer (Thermo Fisher Scientific, Waltham, MA, USA).

## 3. Results and Discussion

### 3.1. Characterizations

**Figure 1:**
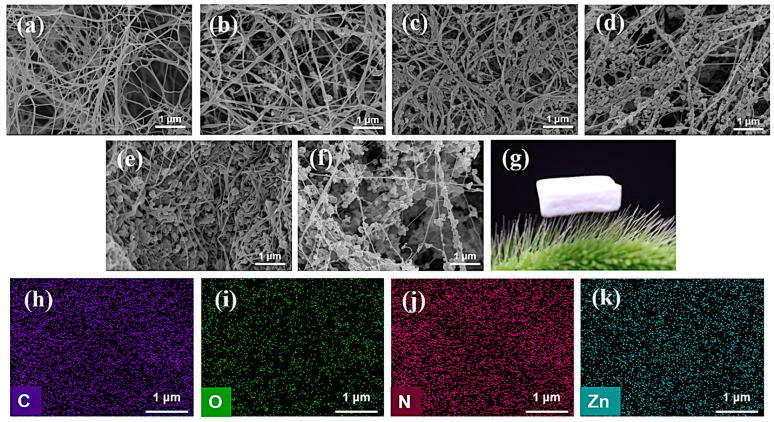
SEM images of (**a**) BC; (**b**) ZIF-8@BC-1; (**c**) ZIF-8@BC-2; (**d**) ZIF-8@BC-3; (**e**) ZIF-8@BC-4; (**f**) ZIF-8@BC-5; (**g**) Photo of ZIF-8@BC-4 placed on foxtail grass; (**h**) Element C; (**i**) Element O; (**j**) Element N; (**k**) Element Zn of energy spectrum analysis for the ZIF-8@BC-4 aerogel.

The morphological and structural characteristics of BC and ZIF-8@BC-X were analyzed through SEM. As illustrated in Figure 1a, BC features a three-dimensional network structure composed of subfibrils with a diameter of about 1.5 nm, which aggregate into 2–4 nm wide nanofibrils through hydrogen bonding. These nanofibrils subsequently self-assemble into nanoribbons measuring 40–60 nm in width, which are further interwoven to establish a BC network [33,34]. Figure 1b–f illustrate the structure of the ZIF-8@BC aerogels, revealing that ZIF-8 aerogels are uniformly distributed across the BC surface. With the increasing content of 2-MI and zinc nitrate, the loading of ZIF-8 onto BC rises progressively, while the BC morphology remains stable. The findings derived from the elemental analysis (Figure 1h–k) further confirm that ZIF-8 aerogels are evenly distributed on the BC surface, showing no evidence of aggregation. Additionally, the resultant ZIF-8@BC-X aerogel exhibits excellent lightweight properties (Figure 1g).

**Figure 2:**
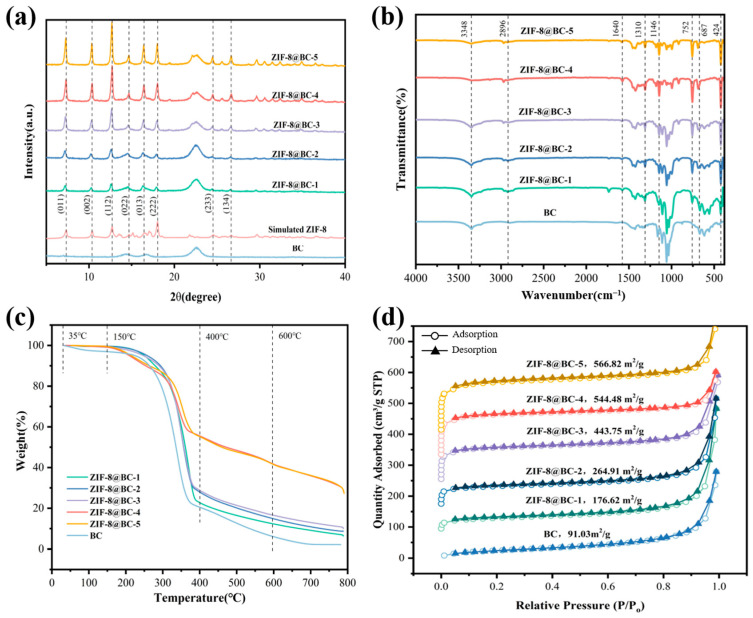
The obtained (**a**) XRD and (**b**) FTIR spectra, along with (**c**) TGA results, as well as (**d**) N_2_ adsorption–desorption isotherms of BC and ZIF-8@BC-X.

According to the XRD patterns illustrated in Figure 2a, combined with the reference pattern of ZIF-8, the diffraction peaks observed at 7.3°, 10.5°, 12.9°, 14.9°, 16.4°, 18.2°, 24.7°, and 26.8° are associated with the (011), (002), (112), (022), (013), (222), (233), and (134) planes of ZIF-8, respectively [35]. The BC spectrum reveals diffraction peaks at 14.4°, 16.8°, and 22.5°, respectively, ascribed to the presence of (1$\bar{1}$0), (110), and (200) planes of cellulose I. The presence of these characteristic peaks in the ZIF-8@BC-X spectrum indicates that ZIF-8 has been successfully incorporated onto the BC surface while preserving its crystal structure. The chemical composition and functional groups of BC and ZIF-8@BC-X were determined using FTIR spectroscopy (Figure 2b). For the case of BC, the characteristic peak at 3348 cm^−1^ is associated with the stretching vibration of O-H. The O-H bending vibration frequency at 1640 cm^−1^ indicates the presence of trace amounts of water. The signal appearing at 2896 cm^−1^ is associated with the stretching vibration of the C-H bonds corresponding to the aliphatic moieties. Importantly, the vibrational bands of polysaccharides in BC are distinguishable at 1160, 1110, 1056, and 1034 cm^−1^ [36]. The ZIF-8@BC-X aerogel exhibits characteristic vibrational bands at 424, 687, 752, 1146, and 1310 cm^−1^, which are consistent with the FTIR data of ZIF-8 [37]. Among them, the fingerprint region at 687 and 752 cm^−1^ was associated with the bending vibration of the methylimidazole ring plane, while the signal at 424 cm^−1^ was ascribed to the bending vibration of the Zn-N bond on the imidazole ring. Furthermore, the stretching vibration related to the C-N bond appears at 1146 cm^−1^, while the overall stretching vibration of the entire methylimidazole occurs at approximately 1310 cm^−1^. These findings confirm that ZIF-8 has been effectively loaded onto the BC surface. Moreover, the peak at 1640 cm^−1^ corresponding to the adsorbed water in BC weakened after synthesis, which indicates that the influence of ZIF-8 on BC is due to their interaction. The XPS spectra further verified the above speculation. In the full-spectrum of BC and ZIF-8@BC-4 (Figure S2), it was observed that the ZIF-8@BC-4 contained C and O elements, while a new N 1s (399 eV) and Zn 2p (1022 eV) peak appeared, confirming that ZIF-8 was effectively deposited onto the BC surface.

The thermogravimetric analysis presented in Figure 2c for BC and ZIF-8@BC-X demonstrated a minor mass loss between 35 °C and 150 °C, ascribed to the evaporation of water or *tert*-butanol retained within the cavities or on the surfaces of BC and ZIF-8@BC-X. BC commenced decomposition at 250 °C. ZIF-8@BC-X displayed decomposition of the organic framework from 150 °C to 600 °C, with the release of organic ligands progressively increasing from 600 °C to 800 °C [38]. Notably, ZIF-8@BC-X exhibited a decomposition curve resembling that of BC from 250 °C to 400 °C, primarily due to cellulose decomposition. The observed increase in weight of ZIF-8@BC-X following thermal decomposition is attributed to the heightened amount of ZIF-8 incorporated into ZIF-8@BC-X. The curves for ZIF-8@BC-4 and ZIF-8@BC-5 partially overlap, indicating that the ZIF-8 loading onto BC may have reached saturation.

It has been demonstrated that ZIF-8 possesses a high specific surface area and nano-scale pore structure, which endows it with strong adsorption properties for pollutants [39]. The N_2_ adsorption–desorption curve results (Figure 2d) show that the ZIF-8@BC-X composite aerogel exhibits a type I curve, with a clear adsorption effect in the low-pressure region where P/P_0_ < 0.1, indicating the presence of a large number of micropores in the aerogel. The pore distribution curve (Figure S3) indicates that the ZIF-8@BC-X composite aerogel maintains the nanoscale porous structure of ZIF-8 (<2 nm) and, together with the pristine BC with micro-scale pores, constitutes the composite aerogel with high specific surface area and multi-level pores. The BET results (as shown in Figure 2d) implied that the specific surface area of ZIF-8@BC-X increased progressively with the rising loading of ZIF-8. Specifically, the specific surface area for BC was 91.03 m^2^/g, while the specific surface areas for ZIF-8@BC-X (ZIF-8@BC-1, ZIF-8@BC-2, ZIF-8@BC-3, ZIF-8@BC-4, and ZIF-8@BC-5) were 176.62, 264.91, 443.75, 544.48, and 566.82 m^2^/g, respectively. The average specific surface area measured 399.32 m^2^/g, which is about 4.4 times greater than that of the pristine BC. The considerable specific surface area and hierarchical porous structure confer a high adsorption capacity to ZIF-8@BC-X.

### 3.2. Adsorption Experiment

**Figure 3:**
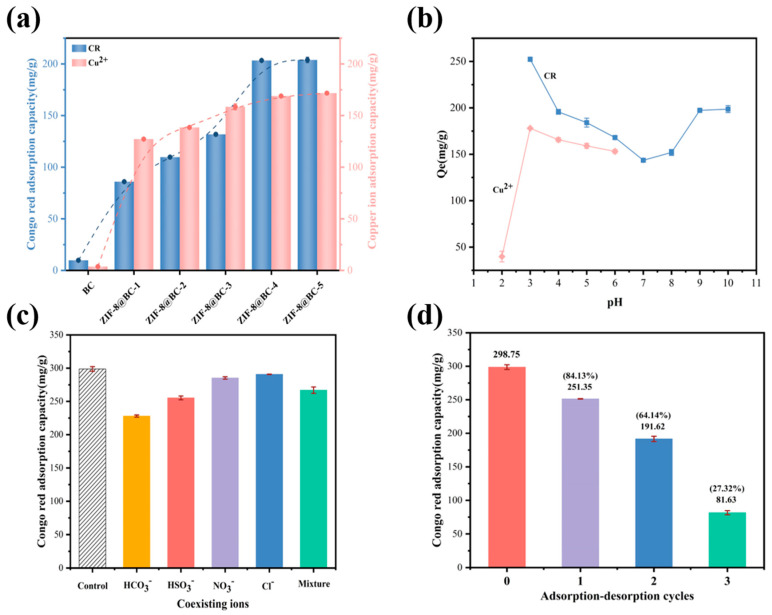
(**a**) Influence of BC and ZIF-8@BC-X on the adsorption effect of CR and Cu^2+^; (**b**) Impact of pH value on the adsorption efficiency of CR and Cu^2+^ by ZIF-8@BC-4; (**c**) Impact of coexisting anions on the adsorption efficiency of CR by ZIF-8@BC-4; (**d**) Recycling test of ZIF-8@BC-4 for CR.

Figure 3a shows the adsorption effect of ZIF-8@BC-X on CR and Cu^2+^. The results showed that BC exhibited poor adsorption performance for CR and Cu^2+^ due to the lack of adsorption sites, recording values of 9.83 mg/g for CR and 3.68 mg/g for Cu^2+^. Compared with that of BC, all the ZIF-8@BC-X samples have stronger adsorption for CR and Cu^2+^, and the adsorption capacity increases with increasing ZIF-8 loading. The adsorption capacities for CR were 85.97, 109.76, 131.82, 203.35, and 203.86 mg/g, respectively, whereas the adsorption capacities for Cu^2+^ were found to be 127.17, 138.48, 158.44, 168.87, and 171.68 mg/g, respectively. The observed result is consistent with existing research; the ZIF-8-loaded natural kapok fiber micromotor can efficiently remove CR [40], and the 3D-printed cellulose/ZIF-8 can effectively remove Cu^2+^ [41]. The adsorption effects of ZIF-8@BC-4 and ZIF-8@BC-5 on CR and Cu^2+^ were comparable. Considering its economic benefits, ZIF-8@BC-4 was selected for subsequent studies, including its adsorption isotherm, kinetics, and recoverability.

#### 3.2.1. Impact of pH Value

The pH of the solution can influence the surface charge, degree of protonation, and number of active sites along with the ionization of the adsorbed substances within ZIF-8@BC composite aerogels [42,43], thereby playing an essential role in the adsorption effectiveness of ZIF-8@BC aerogels. A series of experiments were conducted to evaluate the influence of pH on the adsorption characteristics of ZIF-8@BC-4. Given that Cu^2+^ tends to form a precipitate in alkaline conditions, the pH range for Cu^2+^ was established between 2.0 and 6.0, while the pH value for CR was varied from 3.0 to 10.0. As the pH increased, the adsorption capacity of ZIF-8@BC-4 for CR initially decreased significantly but subsequently increased slowly (Figure 3b). The optimal adsorption capacity was achieved at a solution pH of 3.0. This phenomenon can be attributed to CR being an anionic dye that undergoes protonation as pH varies [44]. The peak adsorption capacity at pH 3.0 may be linked to the electrostatic interactions between the positively charged ZIF-8@BC-4 and negatively charged CR. The removal rate of Cu^2+^ demonstrated a rapid initial increase, followed by a gradual decline as the pH increased (Figure 3b). At a pH of 3.0, the ZIF-8@BC-4 attained maximum adsorption capacity. Below a pH of 3.0, the Cu^2+^ removal rate was notably low, attributable to the competitive adsorption of Cu^2+^ and H^+^, resulting in many hydrogen ions occupying the active adsorption sites designated for Cu^2+^. Additionally, this is associated with the deprotonation and protonation processes of the functional groups on ZIF-8@BC-4. Functional groups such as –NH_2_ and –OH, when protonated, reduce the number of active adsorption sites available on ZIF-8@BC-4, diminishing the affinity between ZIF-8@BC-4 and Cu^2+^ [45]. Consequently, pH 3.0 was identified as the optimal condition for further testing.

#### 3.2.2. Impact of Coexisting Ions on Adsorption

In wastewater, various other ions coexist with dyes, and the existence of these ions may influence the adsorption effectiveness of the ZIF-8@BC composite aerogel on CR. Consequently, to investigate the adsorption selectivity of ZIF-8@BC-4 for CR in the presence of different mixed ions, the adsorption performance was evaluated with coexisting ions (NO_3_^−^, HSO_3_^−^, HCO_3_^−^, Cl^−^) at a concentration of 100 mg/L. The findings presented in Figure 3c indicate that the adsorption capacity of the group containing coexisting ions is similar to that of the blank sample, which lacked additional ions for CR adsorption. The adsorption capacity associated with the blank group was recorded at 298.75 mg/g, while for the coexisting ion group, it was measured at 266.88 mg/g. This finding suggests that the presence of coexisting ions minimally affected the adsorption selectivity of the ZIF-8@BC-4 composite aerogel for CR.

#### 3.2.3. Reusability Test

The reusability of adsorbents is an essential factor for determining their potential for practical applications. To this end, a cyclic utilization test for CR adsorption–desorption was performed using the ZIF-8@BC-4 aerogel, with the results illustrated in Figure 3d. Following the first regeneration cycle, ZIF-8@BC-4 retained 84.13% of its initial adsorption capacity, measuring 251.35 mg/g. After the second regeneration cycle, the adsorption capacity was reduced to 191.62 mg/g, corresponding to a retention of 64.14% of the original capacity. The capacity after the third cycle was recorded at 81.63 mg/g, indicating a significant reduction in adsorption, with only about 27.32% of the initial capacity remaining. Nevertheless, even after three regeneration cycles, ZIF-8@BC-4 maintained a higher adsorption capacity for CR than many other adsorbents documented in the literature [46]. These results suggest that the ZIF-8@BC-X composite aerogel possesses good reusability for CR adsorption. However, the adsorption efficiency of the ZIF-8@BC-X composite aerogel for Cu^2+^ demonstrated poor cycling effectiveness, likely related to the specific mechanism of Cu^2+^ adsorption onto this composite aerogel.

#### 3.2.4. Adsorption Isotherms

**Figure 4:**
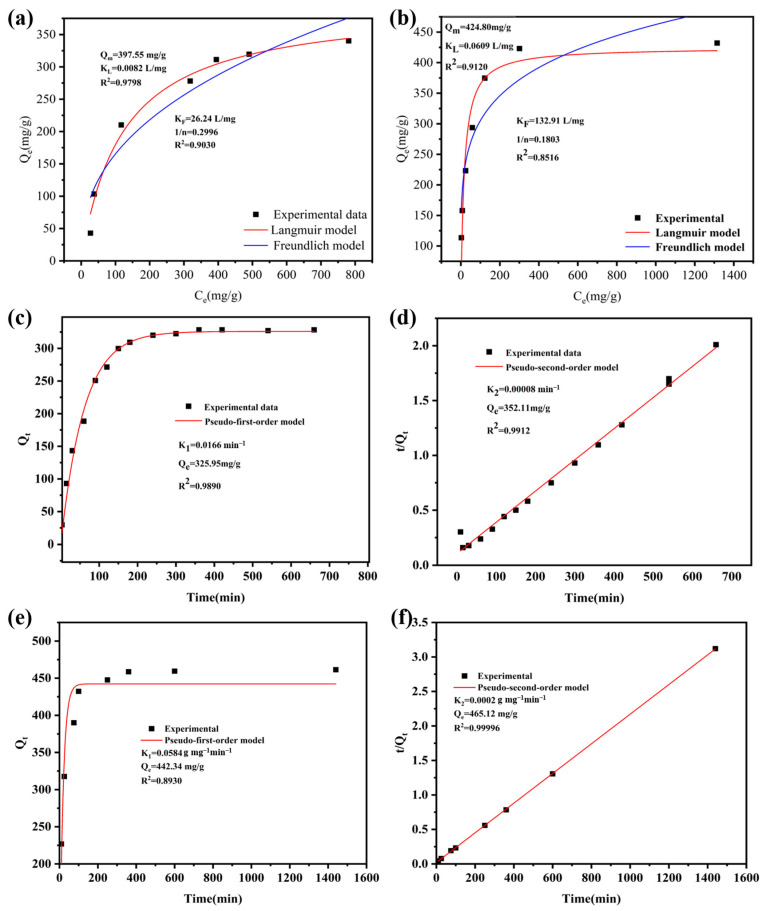
The Freundlich and Langmuir models for the adsorption of ZIF-8@BC-4 for (**a**) CR and (**b**) Cu^2+^; (**c**) PFO kinetic model of CR adsorption on ZIF-8@BC-4; (**d**) PSO kinetic model of CR adsorption on ZIF-8@BC-4; (**e**) PFO kinetic model of Cu^2+^ adsorption on ZIF-8@BC-4; (**f**) PSO kinetic model of Cu^2+^ adsorption on ZIF-8@BC-4.

Adsorption isotherms effectively illustrate the variations in adsorption capacity with different concentrations of adsorbates. To gain a clearer understanding of the interaction mechanism between adsorbents and adsorbates, establishing an appropriate adsorption model is essential [47]. The Langmuir isotherm model posits that the adsorbent’s surface properties are uniform and that only single-molecule adsorption occurs, while the Freundlich isotherm model applies to heterogeneous adsorption surfaces featuring multiple layers of adsorption [48]. Figure 4a presents the isothermal adsorption data for ZIF-8@BC-4 with CR, and Figure 4b shows the isothermal adsorption data for ZIF-8@BC-4 with Cu^2+^. The adsorption capacity of ZIF-8@BC-4 initially increases rapidly with rising concentrations of CR or Cu^2+^ and then gradually rises until a steady state is achieved. This behavior can be linked to the presence of numerous vacancies on the surface of ZIF-8@BC-4, which can quickly adsorb CR or Cu^2+^. However, with increasing solution concentration, the active sites within the vacancies gradually become saturated, leading to no further changes in adsorption capacity. Table 1 lists the parameters of the two isothermal adsorption models. The data indicate that for CR and Cu^2+^, the correlation coefficients (R^2^_CR_ = 0.9798, R^2^_Cu_ = 0.9120) of the Langmuir model are closer to 1, while the correlation coefficients (R^2^_CR_ = 0.9030, R^2^_Cu_ = 0.8516) of the Freundlich model are considerably lower. The Langmuir isotherm model indicated that the maximum adsorption capacities of ZIF-8@BC-4 were 397.55 mg/g for CR and 424.80 mg/g for Cu^2+^. The Langmuir isotherm model more accurately describes the adsorption process of ZIF-8@BC-4 for CR and Cu^2+^, suggesting that the surface of ZIF-8@BC-4 is uniform and that adsorption occurs in a single molecular layer, with all adsorption sites having the same properties and energy levels. The constant 1/n was significantly less than 1 in the Freundlich isotherm model, indicating an uneven surface of the adsorbent; conversely, a value of 1/n approaching 1 signifies relatively uniform binding sites in the material [49]. The 1/n values derived from the analysis in Table 1 are all close to 1, suggesting that ZIF-8@BC-4 acts as a homogeneous adsorbent. Additionally, the value of 1/n is linked to the adsorption strength, with 0 < 1/n ≤ 1 indicating favorable adsorption conditions [50]. This suggests that the ZIF-8@BC-4 composite aerogel is an effective adsorbent for removing CR and Cu^2+^ from aqueous solutions.

Table 2 lists the adsorption characteristics of ZIF-8@BC-4 and other adsorbents for CR and Cu^2+^. The adsorption potential of ZIF-8@BC-4 for CR and Cu^2+^ is much greater than that of the other adsorbents, indicating excellent adsorption capacity. Therefore, it can be used as an ideal adsorbent for the treatment of wastewater containing CR and Cu^2+^.

#### 3.2.5. Adsorption Kinetics

Understanding adsorption kinetics is essential for analyzing the underlying adsorption mechanism. The results of the adsorption of CR and Cu^2+^ on ZIF-8@BC-4 over time are shown in Figure 4c–f. During the initial stage of the reaction, ZIF-8@BC-4 adsorbs rapidly owing to the presence of excessive active regions on the surface of the ZIF-8@BC-4 composite aerogel. Cu^2+^ reaches adsorption equilibrium at about 100 min, whereas CR adsorbs slowly and reaches adsorption equilibrium at about 150 min. Table 1 shows that the adsorption of CR and Cu^2+^ on ZIF-8@BC-4 aligns more closely with a PSO kinetic model, as demonstrated by a higher R^2^ value upon comparison with the PFO model, suggesting that the adsorption rates are primarily governed by chemical interactions [63,64].

### 3.3. Adsorption Mechanism

The adsorption mechanism is influenced by various interactions, with the primary ones being electrostatic interactions and host–guest interactions [65]. The specific type of interaction, however, is contingent upon the surface charge of ZIF-8@BC-4, the functional groups, and the structure of the adsorbate. Chemical characterization and analyses, including FTIR, XRD, EDS, and XPS, were carried out on ZIF-8@BC-4 both before and following the adsorption of CR and Cu^2+^ to investigate the underlying adsorption mechanisms.

**Figure 5:**
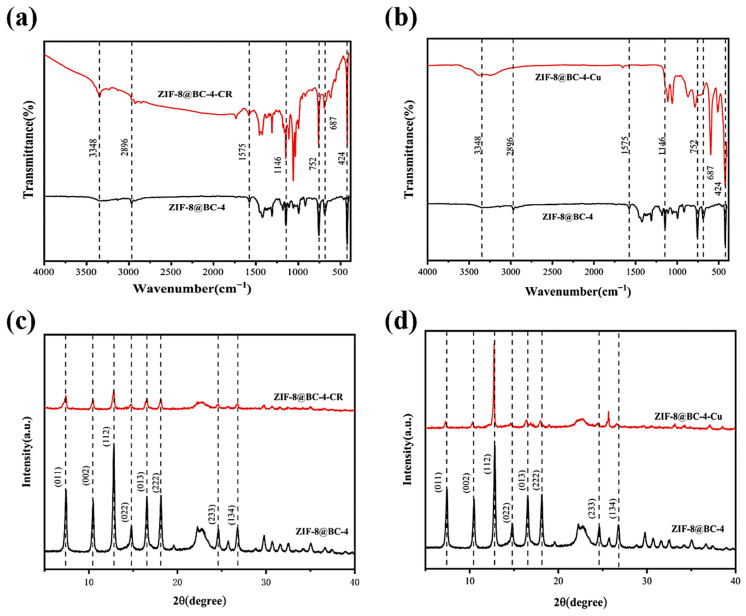
FTIR spectra of ZIF-8@BC-4 prior to and following the adsorption of CR (**a**) and Cu^2+^ (**b**); XRD patterns of ZIF-8@BC-4 before and after the adsorption of CR (**c**) and Cu^2+^ (**d**).

Figure 5a presents the FTIR spectra of ZIF-8@BC-4 prior to and following the adsorption of CR. A substantial amount of CR is adsorbed onto the ZIF-8@BC-4 surface, obscuring the original imidazole characteristic peaks. Following the CR adsorption onto ZIF-8@BC-4, the peak at 3348 cm^−1^ exhibited a significant increase, while the peak at 424 cm^−1^ significantly decreased, potentially due to the hydrolysis of Zn-N in ZIF-8 to Zn-OH and N-H upon exposure to water. Moreover, the O-H stretching vibration (3348 cm^−1^) overlaps with the -NH_2_ stretching vibration peak of CR, suggesting the occurrence of hydrogen bonding between ZIF-8@BC-4 and CR [66]. The intensity associated with the characteristic peak at 1575 cm^−1^ (corresponding to the C=N stretching mode in ZIF-8) displayed a notable decrease, indicating the presence of a π-π bond between the imidazole ring on the surface of ZIF-8 and the aromatic ring in CR. Additionally, a new characteristic signal appeared at 1058 and 617 cm^−1^, ascribed to the vibrations of the S=S and -SO_3_ groups of CR. The XRD spectrum in Figure 5c indicates the noticeable reduction in the characteristic diffraction peaks of ZIF-8, which is in agreement with the FTIR analysis. Figure S4 shows the elemental spectra of O, C, N, and Zn after adsorption of CR. It is indicated that the content of C, O, and N enhanced substantially, which is linked to the increase in elemental energy after the adsorption of CR.

Figure 5b shows the FTIR spectra of ZIF-8@BC-4 before and following the adsorption of Cu^2+^. Following the adsorption of Cu^2+^, ion exchange between Cu^2+^ and Zn^2+^ occurs, altering the structure and composition of the ZIF-8 adsorbent surface and leading to corresponding shifts in the characteristic peaks of imidazole. Furthermore, the decrease in the signal at 424 cm^−1^ may be ascribed to the hydrolysis of Zn-N in ZIF-8 to Zn-OH and N-H after ZIF-8@BC-4 contacts Cu^2+^. The peak at 1650 cm^−1^ denotes the vibrational signal of -NH_2_, which has experienced a slight shift, indicating that the amino groups on the surface of ZIF-8@BC-4 have chemically bound to Cu^2+^. In Figure 5d, it is apparent that the characteristic diffraction peaks associated with ZIF-8 have decreased. This is aligned with the FTIR analysis. Figure S5 shows the elemental spectra of C, N, O, Cu, and Zn after the adsorption of Cu^2+^. In addition, Figure S5 shows that Cu^2+^ is evenly distributed in the ZIF-8@BC-4 adsorbent, confirming that adsorption occurs on and within the framework.

**Figure 6:**
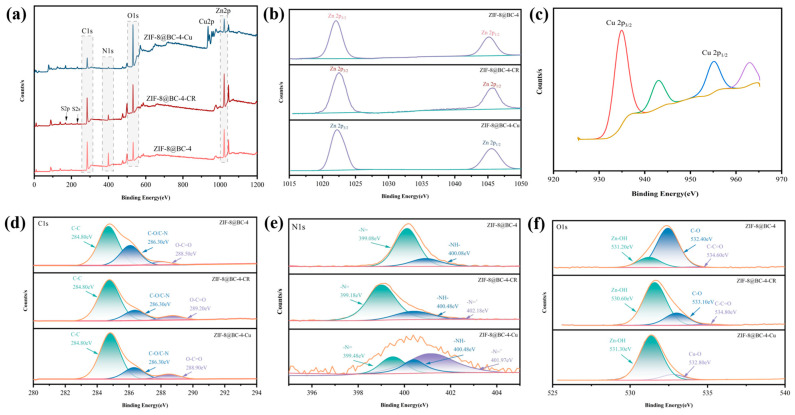
XPS analysis. (**a**) Full spectrum of ZIF-8@BC-4 prior to and following adsorption of CR and Cu^2+^; Zn 2p (**b**), Cu 2p (**c**), C 1s (**d**), N 1s (**e**), and O 1s (**f**) before adsorption of CR and Cu^2+^ for ZIF-8@BC-4.

Figure 6 presents the XPS spectra obtained before and after the adsorption of ZIF-8@BC-4. The characteristic peaks corresponding to O 1s, C 1s, N 1s, and Zn 2p are clearly visible in the full spectra of Figure 6a. Following the adsorption of CR, peaks related to S 2s and S 2p are observed, confirming the successful adsorption of CR. However, the results of XPS spectra indicate that the characteristic peak of Na 1s in the CR is absent in the adsorbed ZIF-8@BC-4, suggesting that the negative sulfonic acid group (-SO_3_-) in the CR interacts with the positive ions on ZIF-8@BC-4 and is adsorbed through electrostatic forces. During this adsorption process, sodium ions should be replaced by positive ions. This result is consistent with the SEM-EDS results in Figure S4. To achieve further confirmation regarding the alterations in the surface chemical state and elemental composition of ZIF-8@BC-4 prior to and following the adsorption of CR, the XPS spectra were analyzed through peak separation fitting. In the C 1s subpeak energy spectrum (Figure 6d), three characteristic peaks of C-C (284.8 eV), C-O/C-N (286.3 eV), and O-C=O (288.5 eV) were identified. After the adsorption of CR, a reduction in the intensity of the C-O/C-N signal was noted, whereas the intensities of the C-C and O-C=O peaks increased, indicating an interaction between ZIF-8@BC-4 and CR. Three characteristic peaks appeared in the O 1s subpeak energy spectrum (Figure 6f), specifically Zn-OH (531.2 eV), C-O (532.4 eV), and C-C=O (534.6 eV). After the adsorption of CR, the peak intensity of Zn-OH significantly increased, indicating the hydrolysis of Zn-N in ZIF-8 into Zn-OH upon contact with water, which aligns with the findings derived from FTIR. Following the adsorption of CR, the Zn-OH signal moved to 530.6 eV, while the C-O peak weakened and shifted to 533.1 eV, which resulted from the shared electron pair bond formed between O atoms and CR occupying the lone pair electrons originally associated with O atoms. Two peaks were observed in the N 1s spectrum (-NH- at 400.08 eV, -N= at 399.08 eV); both shifted to higher binding energy positions (-NH- at 400.48 eV, -N= at 399.18 eV) because the shared electron pair bonds between N and CR occupied the lone pair electrons originally belonging to the N atom. Furthermore, a protonated N atomic group (-N=^+^) with a binding energy of 402.18 eV was produced after CR adsorption, which facilitated the electrostatic interaction between negatively charged CR and the ZIF-8@BC-4.

The Cu 2p peak exists in the spectrum of the adsorbed ZIF-8@BC-4 (as shown in Figure 6a), further indicating that Cu^2+^ is adsorbed on ZIF-8@BC-4. Following the adsorption of Cu^2+^, the Zn-OH peak shifts to 531.3 eV, the C-O peak disappears, and a new peak, Cu-O, emerges at a binding energy value equal to 532.8 eV, indicating that the active sites (Zn^2+^ and -OH) on the ZIF-8@BC-4 surface may chemically bind to Cu^2+^. Both peaks in the N 1s spectrum have shifted to higher binding energy positions (-NH- at 400.48 eV, -N= at 399.48 eV). Meanwhile, due to the in situ doping of positive Cu^2+^, a protonated N atomic group (-N=^+^) with a binding energy of 401.97 eV was generated.

In summary, the adsorption characteristics of the ZIF-8@BC-4 composite aerogel are influenced by both its physical and chemical adsorption mechanisms. As illustrated in Figure S6, under acidic conditions, some N atom groups (-N=) on the surface of ZIF-8@BC-4 are protonated, resulting in a positive charge, while CR possesses negatively charged sulfonic acid (-SO^3−^) groups. Upon contact between the two, strong electrostatic interactions are established. Additionally, a π-π bond is formed between the imidazole ring on the surface of ZIF-8 and the aromatic ring in CR. Furthermore, when ZIF-8@BC-4 comes into contact with water, the Zn-N in ZIF-8 undergoes hydrolysis to form Zn-OH. The interaction between the -OH groups of BC and the -NH_2_ groups of CR, facilitated by hydrogen bonding, significantly enhances the adsorption process. Moreover, an interaction between Zn^2+^ in ZIF-8@BC-4 and the sulfonic acid group (-SO^3−^) in CR may occur, which could be characterized as a coordination bond [67]. Lastly, the SEM-EDS results suggest the possibility of pore diffusion between ZIF-8@BC-4 and CR.

After adsorbing Cu^2+^, the bond formed between the nitrogen atom and Cu^2+^ on ZIF-8@BC-4 involves the nitrogen’s lone electron pair, promoting electrostatic interactions. Second, the ion exchange between Zn^2+^ and Cu^2+^ on ZIF-8 is essential in the adsorption process. Third, Cu^2+^ is adsorbed on the ZIF-8@BC-4 surface through a pair of lone pair electrons, some of which form a coordination bond with N atoms [68]. Fourth, after ZIF-8 comes into contact with water, the Zn-N in it hydrolyzes into Zn-OH, and the -OH may undergo microchemical precipitation with Cu^2+^ [69]. Finally, according to the SEM-EDS results, there may be pore diffusion between ZIF-8@BC-4 and Cu^2+^.

## 4. Conclusions

In this study, ZIF-8@BC composite aerogels were synthesized in situ, demonstrating effective adsorption capabilities for dyes and toxic metal ions. Furthermore, the synthesized ZIF-8@BC composite aerogel displays excellent selectivity and reusability for dyes, achieving an adsorption capacity of 81.63 mg/g for CR even after three regeneration cycles. The proposed binding mechanism between ZIF-8@BC-4 and CR includes electrostatic interactions, π-π bonding, hydrogen bonding, coordination bonding, and pore diffusion, while the mechanism for the interaction with Cu^2+^ includes electrostatic interactions, ion exchange, coordination bonding, microchemical precipitation, and pore diffusion. The ZIF-8@BC composite aerogel holds significant potential for applications in dye and toxic metal adsorption.

## Figures and Tables

**Table 1:** Adsorption isotherms and kinetic parameters of ZIF-8@BC-4 for CR and Cu^2+^.

	Langmuir			Freundlich			PFO			PSO		
	Q_m_ (mg/g)	K_L_ (L/mg)	R^2^	k_F_ (L/g)	n	R^2^	Q_e_ (mg/g)	k_1_ (min^−1^)	R^2^	Q_e_ (mg/g)	k_2_ (g/mg·min)	R^2^
CR	397.55	0.0082	0.9798	26.24	3.3378	0.9030	325.95	0.0166	0.9890	352.11	0.00008	0.9912
Cu^2+^	424.80	0.0609	0.9120	132.91	5.5463	0.8516	442.34	0.0584	0.8930	465.12	0.0002	0.99996

**Table 2:** Adsorption performance of various adsorbents for CR and Cu^2+^.

Pollutants	Adsorbents	Equilibrium Time (min)	*q*_max_ (mg g^−1^)	Specific Surface Area (m^2^ g^−1^)	Average Pore Diameter (nm)	Pore Volume (cm^3^ g^−1^)	Ref.
CR	BCB@PEI	240	393.37	19.744	38.532	0.1416	[51]
	CEC/OSA/2%Ca^2+^	1500	185.43	—	—	—	[52]
	SEB-700	90	185.32	5.98	9.51	0.03	[53]
	PASL	20	293.26	8.81	21.30	46.89 × 10^3^	[54]
	SASL	20	257.07	11.43	21.28	60.79 × 10^3^	
	TASL	20	165.56	15.39	17.29	67.76 × 10^3^	
	BE/CH@Co	480	303	100.5	—	—	[55]
	ZIF-8@BC-4	150	397.55	544.48	—	—	This work
Cu^2+^	PCC/SA	120	287.55	1.18	10.17	—	[56]
	DPMC	120	298.62	—	—	—	[57]
	PVDF-g-G3 PAMAM	200	153.8		—	—	[58]
	BC-LDH	30	85.47	—	—	—	[59]
	SW-700	350	227.273	19.815	—	0.021	[60]
	ZIF-8-EGCG	300	232.97	113.02	7.12	—	[61]
	KGM + DB18C6	60	194	—	—	—	[62]
	ZIF-8@BC-4	100	424.80	544.48	—	—	This work

## Data Availability

Data are contained within the article and Supplementary Materials.

## Supplementary Materials

The following supporting information can be downloaded at: https://www.mdpi.com/article/10.3390/molecules30050982/s1, Figure S1: Schematic diagram of preparation of ZIF-8@BC-X composite aerogel; Figure S2: XPS analysis of BC and ZIF-8@BC-4; Figure S3: Pore distribution of BC and ZIF-8@BC-X; Figure S4: (a–d) Element map of ZIF-8@BC-4 after adsorption of CR; (e) EDS spectra before adsorption of CR by ZIF-8@BC-4; (f) EDS spectra after adsorption of CR by ZIF-8@BC-4; Figure S5: (a–e) Elemental map of ZIF-8@BC-4 adsorption of Cu^2+^; (f) EDS spectra before adsorption of Cu^2+^ by ZIF-8@BC-4; (g) EDS spectra after adsorption of Cu^2+^ by ZIF-8@BC-4; Figure S6: Mechanisms of adsorption of CR and Cu^2+^ on the ZIF-8@BC-X composite aerogel; Table S1: Preparation of ZIF-8@BC-X.
