# Supplementary material for: Green Fabrication of Zinc-Based Metal–Organic Frameworks@Bacterial Cellulose Aerogels via In Situ Mineralization for Wastewater Treatment
Source: Molecules. 2025 Feb 20;30(5):982. doi: 10.3390/molecules30050982 (PMC11901442; doi:10.3390/molecules30050982)
Supplement: Supplementary file 1 [file molecules-30-00982-s001.zip › molecules-3461158-supplementary.pdf]

Green fabrication of zinc-based metal organic Green Fabrication of Zinc-Based Metal–Organic Frameworks@Bacterial Cellulose Aerogels via In Situ Mineralization for Wastewater Treatment

Xinru Liu [1,†], Jie Gu [1,†], Yongqi Cao [1], Liping Tan [1,2,*] and Tongjun Liu [1,*]

[1] Shandong Provincial Key Laboratory of Microbial Engineering, Department of Bioengineering, Qilu University of Technology, Shandong Academy of Sciences, Jinan 250353, China; 10431221262@stu.qlu.edu.cn (X.L.); gujie0218@163.com (J.G.); 10431240818@stu.qlu.edu.cn (Y.C.)
[2] Guangxi Key Laboratory of Clean Pulp and Papermaking and Pollution Control, College of Light Industry and Food Engineering, Guangxi University, Nanning 530004, China
[*] Correspondence: tanliping@qlu.edu.cn (L.T.); tjliu@qlu.edu.cn (T.L.)
[†] These authors contributed equally to this work.

Number of Pages: 8

Number of Figures: 6

Number of Tables: 1

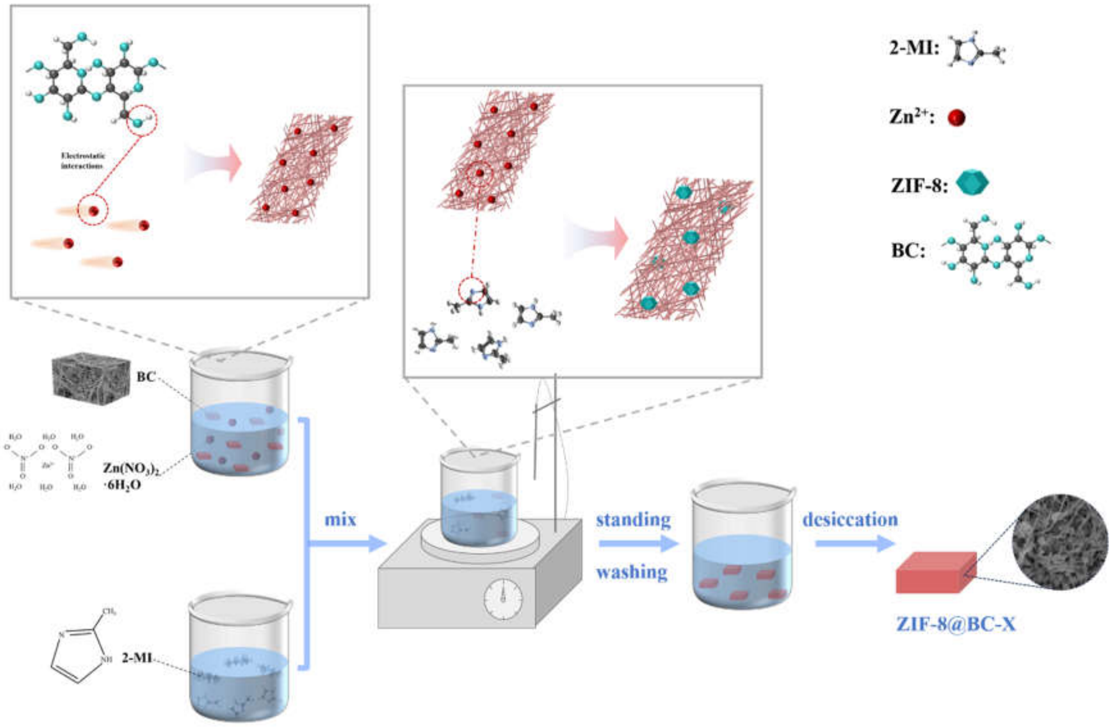

Figure S1. Schematic diagram of preparation of ZIF-8@BC-X composite aerogel.

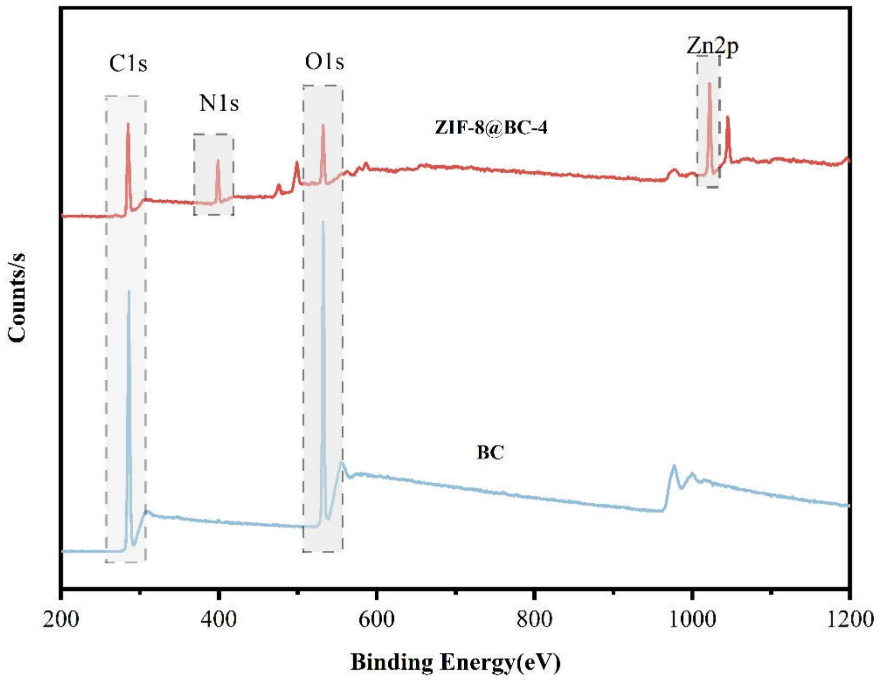

Figure S2. XPS analysis of BC and ZIF-8@BC-4.

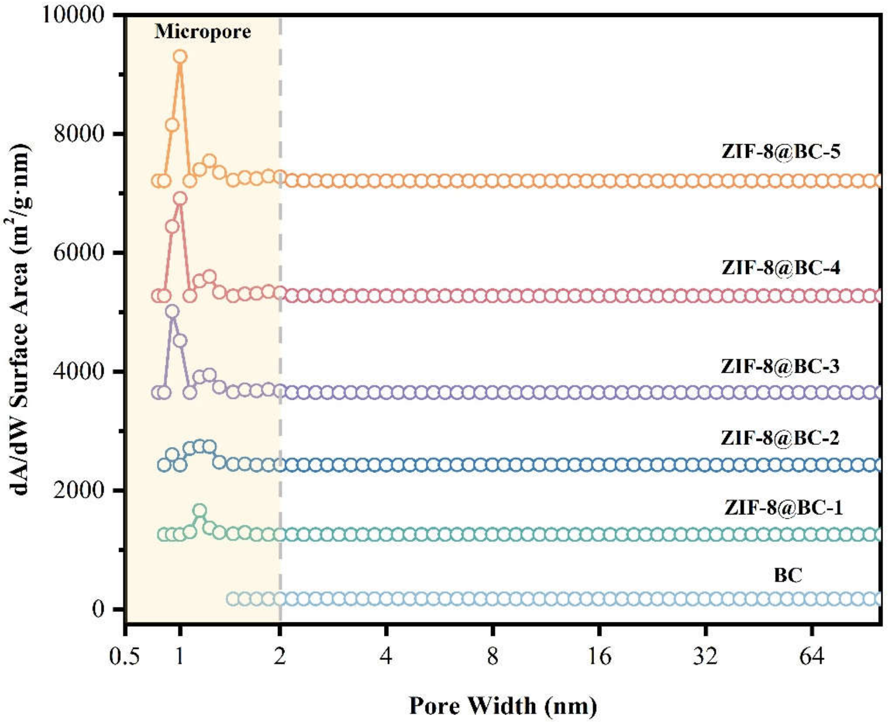

Figure S3. Pore distribution of BC and ZIF-8@BC-X.

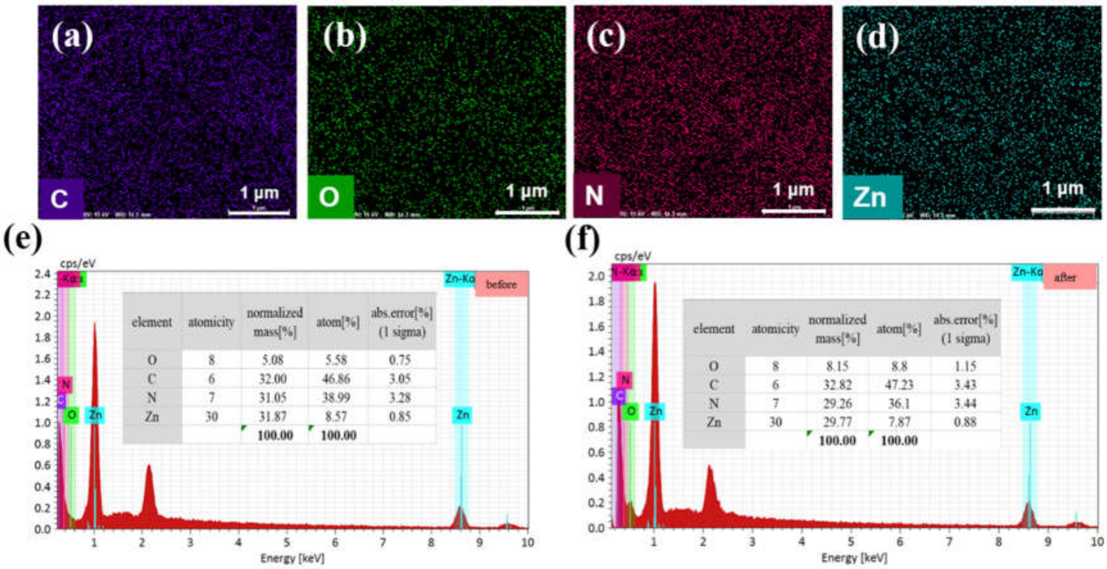

Figure S4. (a-d) Element map of ZIF-8@BC-4 after adsorption of CR; (e) EDS spectra before adsorption of CR by ZIF-8@BC-4; (f) EDS spectra after adsorption of CR by ZIF-8@BC-4.

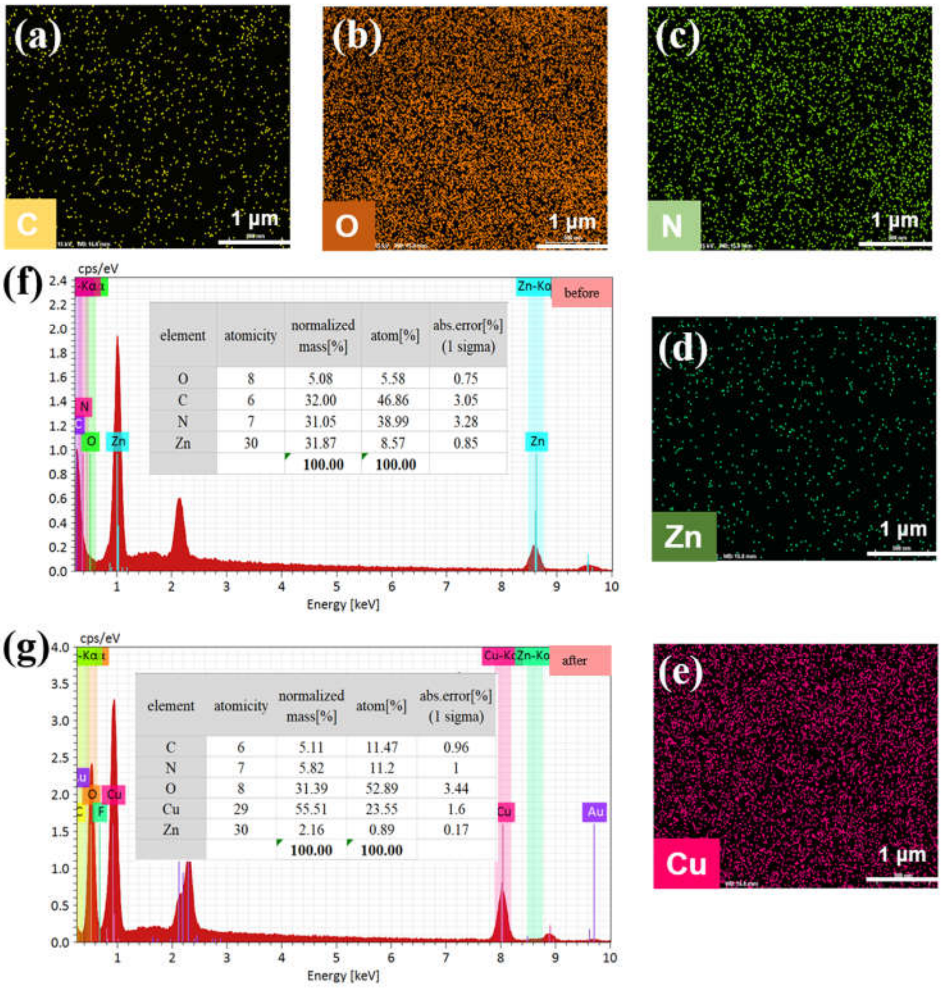

Figure S5. (a-e) Elemental map of ZIF-8@BC-4 adsorption of Cu^{2+}; (f) EDS spectra before adsorption of Cu^{2+} by ZIF-8@BC-4; (g) EDS spectra after adsorption of Cu^{2+} by ZIF-8@BC-4.

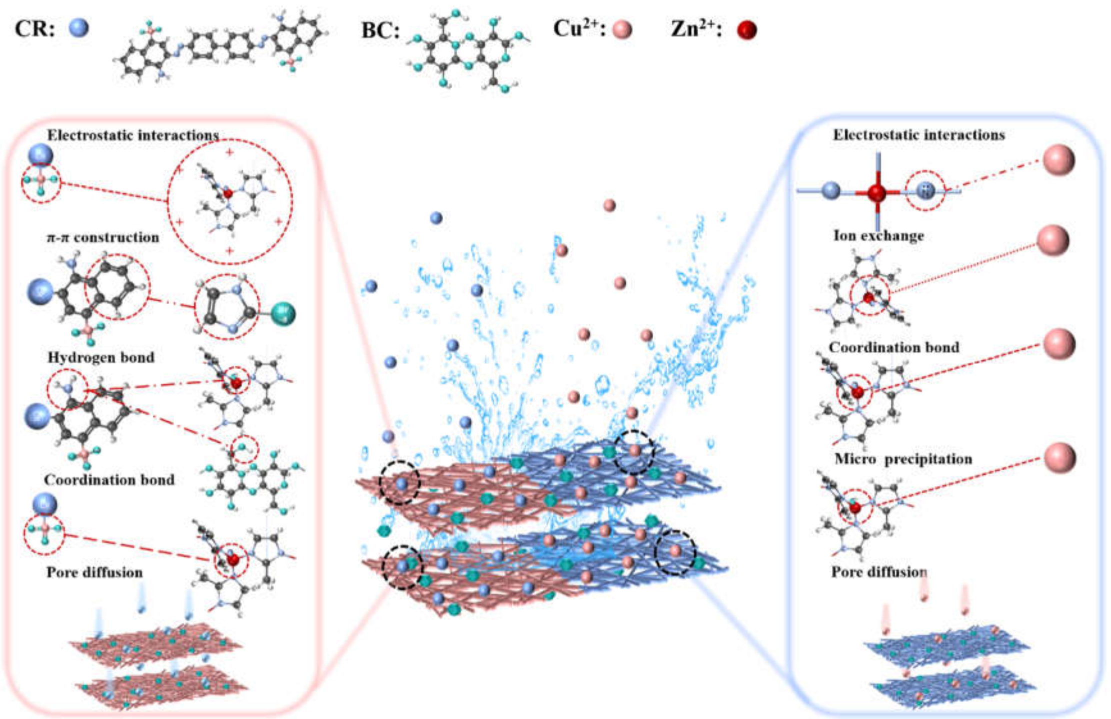

Figure S6. Mechanisms of adsorption of CR and Cu^{2+} on the ZIF-8@BC-X composite aerogel.

Table S1 Preparation of ZIF-8@BC-X.

Samples	Dosage of zinc nitrate (g)	Dosage of 2-methylimidazole (g)
BC	0	0
ZIF-8@BC-1	0.075	0.165
ZIF-8@BC-2	0.15	0.33
ZIF-8@BC-3	0.30	0.66
ZIF-8@BC-4	0.60	1.32
ZIF-8@BC-5	1.20	2.64
